# Treating Duchenne Muscular Dystrophy: The Promise of Stem Cells, Artificial Intelligence, and Multi-Omics

**DOI:** 10.3389/fcvm.2022.851491

**Published:** 2022-03-10

**Authors:** Carlos D. Vera, Angela Zhang, Paul D. Pang, Joseph C. Wu

**Affiliations:** ^1^Stanford Cardiovascular Institute, Stanford University, Stanford, CA, United States; ^2^Division of Cardiovascular Medicine, Department of Medicine, Stanford University, Stanford, CA, United States

**Keywords:** Duchenne muscular dystrophy, cardiomyopathy, iPSC disease modeling, drug testing, single-cell technology, artificial intelligence

## Abstract

Muscular dystrophies are chronic and debilitating disorders caused by progressive muscle wasting. Duchenne muscular dystrophy (DMD) is the most common type. DMD is a well-characterized genetic disorder caused by the absence of dystrophin. Although some therapies exist to treat the symptoms and there are ongoing efforts to correct the underlying molecular defect, patients with muscular dystrophies would greatly benefit from new therapies that target the specific pathways contributing directly to the muscle disorders. Three new advances are poised to change the landscape of therapies for muscular dystrophies such as DMD. First, the advent of human induced pluripotent stem cells (iPSCs) allows researchers to design effective treatment strategies that make up for the gaps missed by conventional “one size fits all” strategies. By characterizing tissue alterations with single-cell resolution and having molecular profiles for therapeutic treatments for a variety of cell types, clinical researchers can design multi-pronged interventions to not just delay degenerative processes, but regenerate healthy tissues. Second, artificial intelligence (AI) will play a significant role in developing future therapies by allowing the aggregation and synthesis of large and disparate datasets to help reveal underlying molecular mechanisms. Third, disease models using a high volume of multi-omics data gathered from diverse sources carry valuable information about converging and diverging pathways. Using these new tools, the results of previous and emerging studies will catalyze precision medicine-based drug development that can tackle devastating disorders such as DMD.

## Introduction

Duchenne muscular dystrophy (DMD) is a lethal genetic disorder, primarily characterized by muscle deterioration and wasting. Ultimately, the majority of DMD patients succumb to cardiac and respiratory complications (1). DMD is caused by mutations in the X-chromosome-linked DMD gene that codes for the dystrophin protein, which is an important component of muscle cells' cytoskeleton. Mutations in DMD generally result in large deletions of the dystrophin protein that reduce structural integrity in muscle cells (2). Loss of dystrophin heavily disrupts the connection between the inner cytoskeleton and the extracellular matrix, known as the dystrophin associated protein complex, leading to structural and functional abnormalities in these mechanically active muscle cells (3). As DMD progresses, additional cell functions are impacted (e.g., disrupted calcium regulation, accumulation of reactive oxygen species, poor mitochondrial energetics, etc.). Muscle cells try to adapt to the disrupted processes (4), but can quickly overcompensate, causing the cell to transition from a stressed to a destabilized state; at this point it begins to secrete inflammatory cytokines, activates fibrosis, and ultimately dies (5). As more cellular processes are disrupted, more evidence of cellular dysfunction is exhibited that can be detected in the form of higher levels of serum biomarkers like creatine kinase and myoglobin as signs of muscle wasting, and TNF-α, IFN-γ, IL-5 and IL-6 as signs of chronic inflammation (6–9). Therapeutic efforts for DMD are therefore divided between targeting (i) the underlying cause of DMD, loss of dystrophin, or (ii) targeting a secondary pathology (10).

DMD is a rapidly progressing disease: it presents between the ages 2–5, loss of ambulation can happen by age 12, and premature death can happen by age 25–30 (1). The guidelines for managing DMD include recommendations on nutrition, physical therapy, and cardiovascular health that can help slow down disease progression (11), but there is currently no cure for the disease. Emerging therapies are targeting the underlying loss of dystrophin via several ideas like transcriptionally inducing the production of more dystrophin, utilizing oligonucleotides to promote exon-skipping of the mutant region, and genome editing the mutant exon, all with the goal of producing a healthy dystrophin molecule (12–14). All these promising strategies are currently being evaluated, but they are not ready to provide current DMD patients with useful options to delay pathologic onset. Furthermore, fixing the dystrophin issue would not spontaneously fix all the incurred muscle damage, and thus some additional therapy will likely be needed to achieve healthy skeletal muscle and cardiac function. Many of the current interventions target the compensatory processes that contribute to muscle inflammation and oxidative stress, but are able to only mildly decelerate loss of ambulation and cardiomyopathy (5, 13). The information gathered through decades of pre-clinical, clinical, and pharmaceutical studies has provided a strong foundation for understanding and treating DMD, and can be used to identify other targets; additionally, the information gathered in clinical trials for any compound can inform on efficacy for these chemicals. Clinical studies have also helped us establish checkpoints for disease progression from early-stage to end-stage, allowing us to predict disease progression and informing treatment.

**Figure 1:**
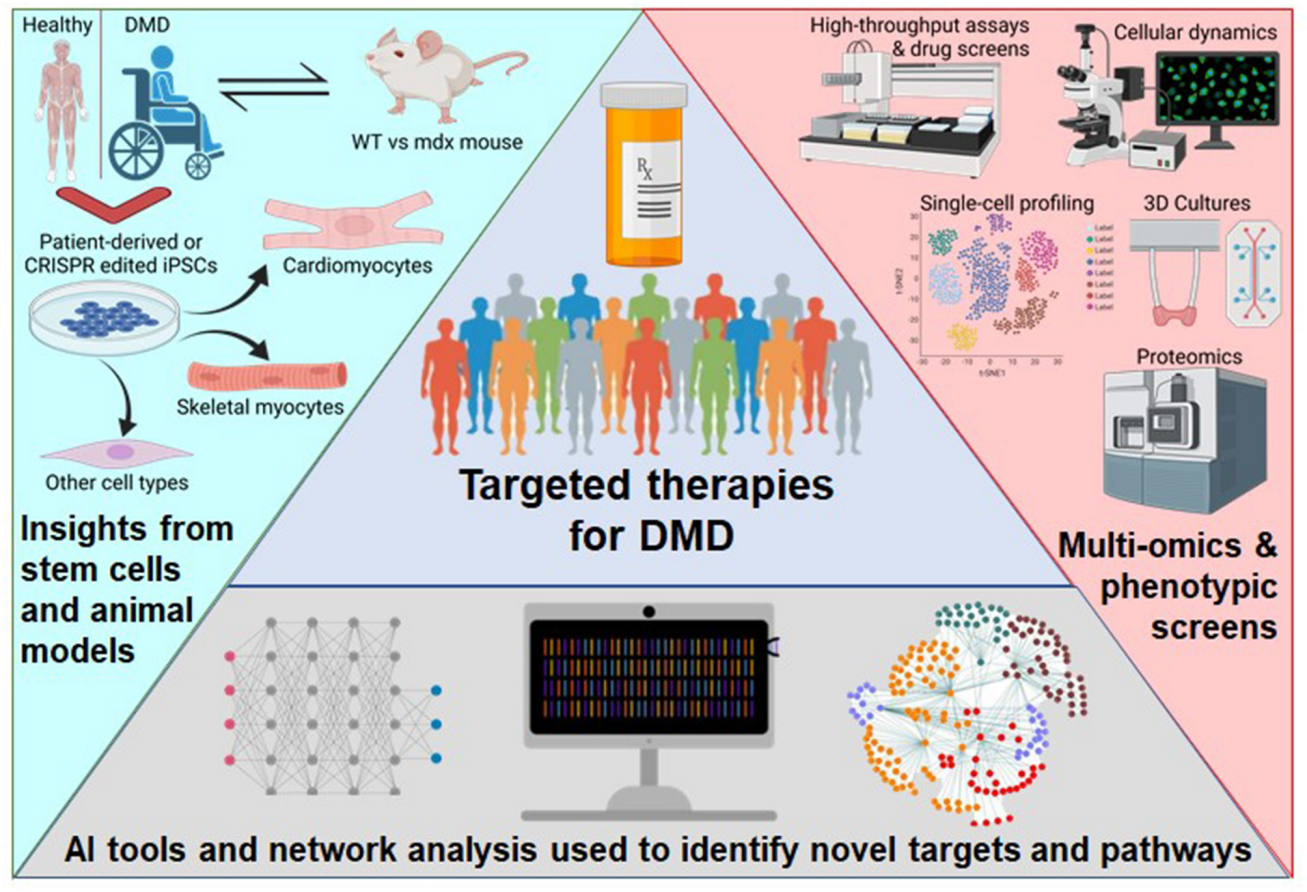
Schematic of the approaches to broaden the therapeutics available to DMD patients. Patient-derived or genome-edited iPSCs provide a human model for disease specific modeling of various cell types and benefits from previous knowledge using the mdx mouse. Newer experimental technologies are detailing the intricacies of complex phenotypes. Next-generation computational tools are enabling high-dimensional analysis of multi-omics data. This figure was created with BioRender.com.

The advent of combining several new approaches holds promise for better and more specific treatments of DMD in the future (Figure 1). First, stem cell-based studies can investigate cellular processes and the effects of different treatments on DMD-afflicted muscle cells without risk to patients. Second, the evolving AI tools could be used to perform high dimensional drug screening with more efficiently streamlined analysis. Third, multi-omics approaches allow the synthesis of information from diverse sources and enable a more holistic understanding of the mechanisms underlying the disease. In this review, we will discuss all three of these approaches, their recent applications, and their potential.

## Human Stem Cells Provide Cell Type- and Disease-Specific Insights

Both DMD-like mouse models and DMD patient samples have yielded mechanistic insights by comparing differential expression of wild-type (WT) and healthy human tissues, pointing to potential pharmaceutical targets (16–18). The mdx mouse has been a standard pre-clinical model for DMD for deciphering how these markers interact and contribute to muscle wasting (19). It has also been extensively used to test the efficacy of the current interventions for DMD (20). However, the reliability of animal models in biomedical research has been questioned due to issues concerning physiological context, species specificity, and clinical relevance (21). An mdx mouse study noted a delayed onset of cardiomyopathy, as opposed to humans which is the main cause of death (22). Even with their contributions to our understanding of the disease, better models that recapitulate the phenotype are necessary (23). By utilizing induced pluripotent stem cells (iPSCs), human disease models hold substantial promise for the clinic, because one can study patient-specific pathologies in multiple different tissues and cell types (24, 25). The use of human iPSCs, in which somatic cells can be reprogrammed to a pluripotent state by introducing four key transcription factors (Oct4, Sox2, Klf4, and c-Myc) and then be chemically programmed to become a cell type of interest (25, 26), has been a ground-breaking technology that promises to treat an entire spectrum of intractable diseases including DMD.

A current effort to directly correct the dystrophin deficiency in DMD is being piloted in the mdx mouse using CRISPR/Cas9 to edit the genetic defect as a therapeutic strategy (15, 27). These efforts have generated a single-cell atlas of skeletal muscle from a dystrophic mouse model that covers both its diseased-state and a CRISPR-corrected state (28). Many known differential expression changes in metabolism, inflammation, and regulatory networks were recapitulated. Moreover, the crosstalk between skeletal myocytes, endothelial cells, macrophages, and various other cell types was explored. In addition, Chemello et al. were able to analyze diverse transcriptional programs stemming from various nuclei, which is important considering that skeletal muscle can house several myonuclei per cell. These spatially defined tissue studies using single-cell RNA-seq can show how different cell types could be targeted for their modified state. Even with the innovation and excitement for spatial transcriptomics studies, there still are trade-offs in utilizing these experiments for mechanistic inference (29). Depending on the question, if a tissue is too large, certain information could be missed if multiple spaces are not covered or considered for analysis, leading to biases. In addition, processing different samples using different tissue separation or cell isolation protocols can create significant variations in the experimental results, which is why there is much interest in automation. Future advances in spatial transcriptomics may overcome some of the present limitations of these experiments (30). Studies utilizing iPSCs can be designed for characterizing the disease-state phenotype of various cell types and testing for drugs.

Recently, human stem cell models have been utilized to study DMD. For example, by identifying issues with cell fate during differentiation, studies have highlighted new potential therapeutic targets (31, 32). There are other examples of how stem cells were successfully deployed to better understand DMD (14, 33). DMD patient-derived iPSCs were used to show accelerated telomere shortening that is seen in DMD patients' heart muscle cells (34). In yet another example, a recent study combined data from both the mdx mouse and DMD patient-derived iPSC-cardiomyocytes to show the overlap in phenotypes between both species and also how both species' cells react to adrenergic receptor stimulation with an agonist (isoproterenol) and antagonist (beta-blocker) (35). This is important because it supports the use of iPSC-derived models as a proper tool for characterizing common DMD treatment effects, just as the mdx mouse has been used for decades. Another recent study characterized the functional effects of known Chinese herbal medicine components on DMD iPSC-cardiomyocytes, highlighting the potential of stem cells for drug discovery (36). This work showed that the anti-oxidant effects of these herbal compounds can be effective at reducing oxidative stress. In a recent review of DMD therapeutics, a meta-analysis of gene expression comparisons of other DMD samples showed that extracellular matrix (ECM) and cell-to-cell interaction molecules are prime targets for reversing late-stage DMD, which makes logical sense because to restore ambulation, the musculature would need to be strengthened (10). Stem cells give us the ability to generate and characterize the different cell types affected by DMD, as well as the ability to perform high-throughput drug screens, paving the way toward patient-specific treatment programs.

## AI Facilitates High-Dimensional Analysis of Large DMD Datasets

Recent advances in omics technologies have led to substantial data generation for disease modeling. However, the wealth of information has resulted in a predictable challenge: how to aggregate and interpret disparate data types, such as transcriptomic, epigenomic, functional, and textual data, which may be distributed across siloed databases (37). Advances in AI have led to models that perform automated text processing and comprehension. Additional AI models can process high-dimensional, non-linear omics data and learn new features about the data that would not be obvious using traditional analysis (38). Simultaneously, progress in computational hardware has increased the ability of researchers to develop and train models to aggregate, analyze, and make predictions using very large datasets. An emerging benefit of using “big-data” algorithms on biomedical information is the ability to tailor treatment programs for individual patients. Information obtained from a person's genome is becoming more reliable and pharmaceutically applicable every day (39). However, designing a specific therapy still requires precisely matching the correct treatment with the unique underlying defect and health state of the patient. The competitive pace of the computational market is driving the current efforts in biotech and pharma to be in position to produce innovative therapies.

Natural language processing (NLP), a machine learning tool with the capability to process text to achieve automated comprehension, translation, and generation, is perhaps one of the most critical AI tools for biomedical research. Initially, NLP primarily relied on recurrent neural networks as the main model of choice. However, recurrent neural networks were limited in the size of the datasets they could train on, and therefore delivered limited performance on clinical and biomedical text comprehension (40). Transformers and attention-based language models have reshaped the landscape of NLP by bypassing existing limitations of recurrent neural networks (41). Importantly, transformers are capable of handling larger datasets than seen before and delivering greater text comprehension, both of which are required to discover novel biological mechanisms and therapeutics from existing biomedical literature and databases (42). When applied to DMD, these recent advances in NLP can query large amounts of DMD-related text and databases to identify key words of interest, annotate DMD with additional clinical phenotypes, molecular pathways, and protein-protein interactions. In drug development, NLP is capable of extracting biological and chemical molecules from existing literature that may interact with DMD pathways and have therapeutics effects.

**Figure 2:**
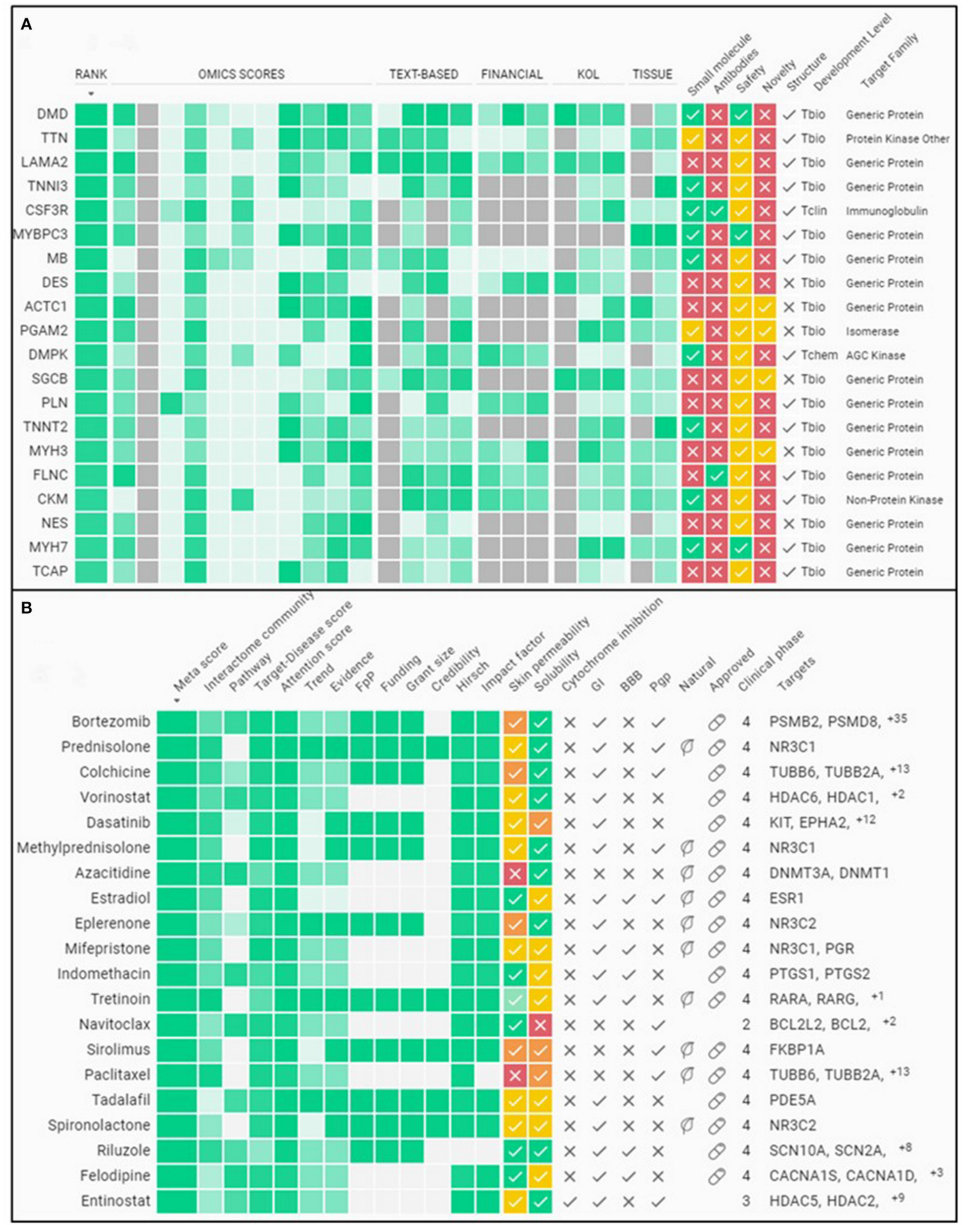
Sample meta-analysis from an A.I. driven therapeutics platform. **(A)** Summary of some of the top gene targets associated to DMD. **(B)** Summary of some of the top compounds associated to DMD for therapy. The evidence for classifying these molecules as the top targets is gleaned from various public databases plus the funding and publications landscape using PandaOmics (http://pandaomics.com/).

As an example, Insilico Medicine has been developing an AI-driven platform that combines multiple datasets from various sources to classify therapeutic targets by their disease relevance, “druggability,” and clinical trajectory. One of the tools is called PandaOmics, which uses deep-learning and cloud-stored data to enable researchers to search information on diseases and their drug targets. The OMICs-sourced analysis of disease relevance is derived from Genome Wide Association Studies, Transcriptome Wide Association Studies, Online Mendelian Inheritance in Man, and the Library of Integrated Network-Based Cellular Signatures, to name a few data bases. For example, PandaOmics combs through clinical trial reports, grant applications, and publications data stemming from the labs mostly associated with the study of a specific disease or compound. This can help inform the larger community, including regulatory agencies, investors, and start-ups, on the basic importance of a target. Their proprietary pathway analysis approach uses iPANDA (in silico Pathway Activation Network Decomposition Analysis) to infer pathway alterations and find significant targets (43). Microarray expression data of skeletal muscles from DMD patients compared to healthy controls are used to quantify common transcriptional changes (44–47). Figures 2A,B illustrate a sample output of a PandaOmics meta-analysis showing some known disease targets and compounds used to treat DMD from the pre-clinical and clinical research stages to full approval for use. Thus, the advent of AI, in particular NLP, allows researchers to aggregate and unearth DMD mechanisms and potential therapeutics that was previously impossible due to the monumental size and siloed nature of existing DMD literature and databases.

### Emerging DMD Multi-Omics Analysis Show Concord and Complexity

Multi-omics experiments for mechanistic insights of DMD have been performed on both animal models (e.g., mdx mouse) and human tissues (e.g., muscle biopsies), thus significantly increasing the diversity of datasets available for analysis. One of the earliest studies into the differences between normal and DMD skeletal muscle transcriptomes was performed on the hind limb and diaphragm muscles of mdx mice compared to control mice (48). This study reported several mechanisms of DMD, including differential expression of IGF-II, NF-kB, SERCA1, RYR1, α-tubulin, and collagens, among many others. A follow-up study comparing mRNA from the gastrocnemius muscle of mdx vs. control mouse used an array analysis of >12,000 genes (49). This study was in agreement with previous reports confirming differential expression of myogenin, α_2_-tubulin, lysozyme M, and myostatin, among others. It also reported upregulation of both IGF-I and IGF-II in dystrophic muscles, but discovered inhibitory IGF-binding proteins and regulators were also increased, thus counteracting the potential beneficial effects of their upregulation. This demonstrates an example of which analyses of multiple datasets are necessary to provide deeper understanding of mechanistic insights. More importantly, Bakay et al. compared their study with human DMD mRNA analyses (49). This revealed notable differences between the transcriptomes of the mdx mouse and human samples, including discordant directional changes in mRNA for myogenin, guanidinoacetate methyltransferase, calponin, and mast cell chymase.

Since these early investigations on the transcriptomic differences, subsequent studies further built upon these mechanistic insights using other omics approaches. For example, studies into microRNAs and chromatin changes revealed the importance of nitric oxide and its associated pathway in DMD pathogenesis (50, 51). Proteomic analysis revealed that Bromodomain and extra-terminal domain (BET) protein BRD4 is significantly increased in the mdx mouse due to direct association to chromatin regulatory regions of the NADPH oxidase subunits (52). Epigenomic analysis, including that on histone acetylation of H3K14 and H3K9 and DNA methylations of Notch1, has revealed its role in regulating satellite cell fate during skeletal muscle regeneration and its dysregulation in DMD (53). Additional chromatin studies also revealed that nuclear pore protein Nup153 associates with chromatin and regulates cardiac gene expression in mdx hearts (54). Recent studies began looking more comprehensively into multi-omics analysis of mdx mouse and human iPSCs. These studies found discrepancies between different omics, such as only a 53% agreement in fold-change data between the proteome and transcriptome in mdx mice (55). Nonetheless, multi-omics studies continue to be a valuable approach in elucidating disease mechanisms as demonstrated by their ability to provide insight into early developmental manifestations of DMD in iPSC models of skeletal muscle differentiation (32). A main challenge remains in integrating these numerous multi-omics datasets into something more precisely meaningful and individually translatable for the patients.

## Summary

Discovering, developing, and delivering targeted therapeutics requires substantial support and collaboration from both academia and industry. It also requires interdisciplinary studies and combinations of skillsets to enable progress. Much is said about how interdisciplinary research is the proper approach to tackling complex diseases even if it also brings challenges in communication and prioritization (56, 57). These challenges can spill over to therapeutics studies where we evaluate how the data we generate correlate with seemingly disconnected sets and how certain factors influence the phenomena we are studying. Machine-learning may not be a new concept, but the influence it has had on biological studies in the past decade is founded on the many novel insights it has generated into how we visualize biological processes and problems (58). The field of DMD has been enriched by advancements in AI and deep learning that now make it possible to aggregate, interpret, and visualize high volumes of high-dimensional and non-linear datasets.

Theoretically, by combining better models of disease risk and severity with detailed analysis of different compounds, we will significantly improve our ability to treat and reduce cardiomyopathy-related deaths. Furthermore, as we approach the goal of fixing the underlying pathological mechanism of diseases like DMD, we must remember to rehabilitate and regenerate healthy tissues to counter persistent dysfunctions and avoid long-term hauling of these issues. To realize these precise therapies will require a convergence of data acquired from state-of-the-art tools and the prioritization of different research agencies and institutes. This will undoubtedly change business models for pharmaceutical and biotech companies, because this will involve a much greater role for drug repurposing, intellectual property disputes and bargains in the coming years. Moreover, on the scientific front, as the quality of analytical tools increases, we will also need the data to be reproducible and of higher quality. The tools we utilize to study cells from different tissues and lineages like iPSCs are becoming ever more reliable and sustainable with single-cell analysis being the standard. Some advances in AI are providing visually interesting graphics that link disease targets to compounds with clinical context. Multi-omics analysis is highlighting the different pathways a disease can take. These developments hold a great potential for improving healthcare by providing clinical researchers with accurate, high-resolution disease-models that will help illuminate the paths to improving patient outcomes.

## Author Contributions

CV wrote the manuscript. AZ, PP, and JW revised the manuscript and provided critical input. All authors contributed to the article and approved the submitted version.

## Funding

This work is supported by National Institutes of Health grants R01 HL126527, R01 HL130020, R01 HL123968, and R01 HL141371 (JW). Due to space limitation, we apologize in advance for not being able to include all references on this topic.

## Conflict of Interest

JW is a co-founder of Greenstone Biosciences. The authors declare that the research was conducted in the absence of any commercial or financial relationships that could be construed as a potential conflict of interest.

## Publisher's Note

All claims expressed in this article are solely those of the authors and do not necessarily represent those of their affiliated organizations, or those of the publisher, the editors and the reviewers. Any product that may be evaluated in this article, or claim that may be made by its manufacturer, is not guaranteed or endorsed by the publisher.
